# Cerebral Venous Sinus Thrombosis in an Immunocompetent HIV Patient

**DOI:** 10.7759/cureus.13694

**Published:** 2021-03-04

**Authors:** Amandeep Rakhra, Luke E Kiefer, Kiran R Busireddy, Rammohan Sankaraneni

**Affiliations:** 1 Internal Medicine, Creighton University School of Medicine, Omaha, USA; 2 Neurology, Creighton University School of Medicine, Omaha, USA

**Keywords:** cvst, vte, hiv, cerebral venous sinus thrombosis, thrombosis, venous thromboembolism, cerebral venous thrombosis, case report, thrombotic coagulopathy, cerebrovascular accidents

## Abstract

Cerebral venous sinus thrombosis (CVST) is an uncommon manifestation in patients with the human immunodeficiency virus (HIV) due to the virus’s prothrombotic state. Our case involves a 41-year-old Hispanic male with a past medical history of HIV on bictegravir/emtricitabine/tenofovir/alafenamide (Biktarvy), hyperlipidemia, post-traumatic stress disorder, hypogonadism with the cessation of testosterone injections one month prior, and generalized anxiety disorder who presented with retro-orbital headache, intermittent bilateral blurry vision, and flashing lights in the lower lateral left eye for one week. Vitals signs and laboratory studies were within normal limits aside from new iron deficiency anemia. Neurological exam was unremarkable. Computed tomography (CT) of the head showed evidence of a subacute cerebral infarct with hemorrhagic transformation in the right superior parietal lobe. Magnetic resonance imaging (MRI) of the brain with contrast revealed a small thrombosed cortical vein with surrounding hemorrhage and edema in the same location, in addition to a partial thrombosis of the adjacent superior sagittal sinus, which was confirmed by magnetic resonance venogram (MRV). Although cerebral angiography was performed, no intervention was attempted for the partially occluded sagittal sinus. HIV viral load was undetectable with a robust cluster of differentiation (CD) 4 count on therapy. The patient was treated with strict blood pressure control, a statin, and a heparin drip. He remained stable and was discharged on enoxaparin injections with bridging to warfarin. In summary, appropriate lab testing, imaging, and high clinical suspicion are required for proper diagnosis and treatment of venous thromboembolism (VTE) or CVST in an HIV-positive patient.

## Introduction

HIV is an infectious process resulting in immunosuppression of its host by the destruction of T cells. It is typically known for causing a variety of opportunistic infections; however, its prothrombotic state secondary to systemic inflammation is less widely recognized. Patients may develop deep vein thromboses (DVTs) in the setting of HIV, and less frequently present with cerebral venous sinus thrombosis (CVST). Furthermore, venous thromboembolisms (VTEs) are uncommon in immunocompetent hosts, usually requiring cluster of differentiation (CD) 4 levels of <200 cells/millimeter^3^ to occur [1]. It is unclear why there is a predilection of certain areas in the body for VTEs in the setting of HIV, but it is important to keep the possibility of cerebral thrombosis in mind should atypical neurological symptoms be present.

## Case presentation

A 41-year-old Hispanic male with a past medical history of HIV on bictegravir/emtricitabine/tenofovir/alafenamide (Biktarvy), hyperlipidemia, post-traumatic stress disorder, hypogonadism with the cessation of testosterone injections one month prior, and generalized anxiety disorder presented with acute headaches with intermittent visual disturbances for one week. His headache was described as retro-orbital with extension to bilateral temporal regions. He complained of several months of intermittent bilateral blurry vision and recent 'flashing lights' disturbance in the lower lateral quadrant of the left eye for one week. In addition, he reported left eye visual disturbance described as a 'lens closing in'. Symptoms were associated with lightheadedness without dizziness, nausea, or vomiting. Episodes of symptoms lasted minutes at a time and occurred multiple times a day.

Family history was significant for cerebral vascular accident (CVA) in two grandparents, both at older ages. He denied any personal or family history of VTEs. Past surgical history was noncontributory and a review of systems was unremarkable. The patient denied smoking and intravenous drug use but affirmed alcohol consumption.

Vitals signs included an oral temperature of 98.1 degrees Fahrenheit, a blood pressure of 148/90 millimeters of mercury (mmHg), a heart rate of 92 beats per minute, and a respiratory rate of 18 breaths per minute with oxygen saturations above 90% on ambient air. Although symptomatic in the preceding days, the patient was asymptomatic at evaluation. Subsequent physical examination, including a complete neurological exam, was unremarkable. Laboratory studies were within normal limits, aside from new iron deficiency anemia (IDA) with hemoglobin 13.2 mg/dl and a normal hematocrit level. His latest HIV viral load was obtained nine months prior and was undetectable, with a CD4 count of 940 cells/millimeter^3^ (cells/mm^3^).

**Figure 1:**
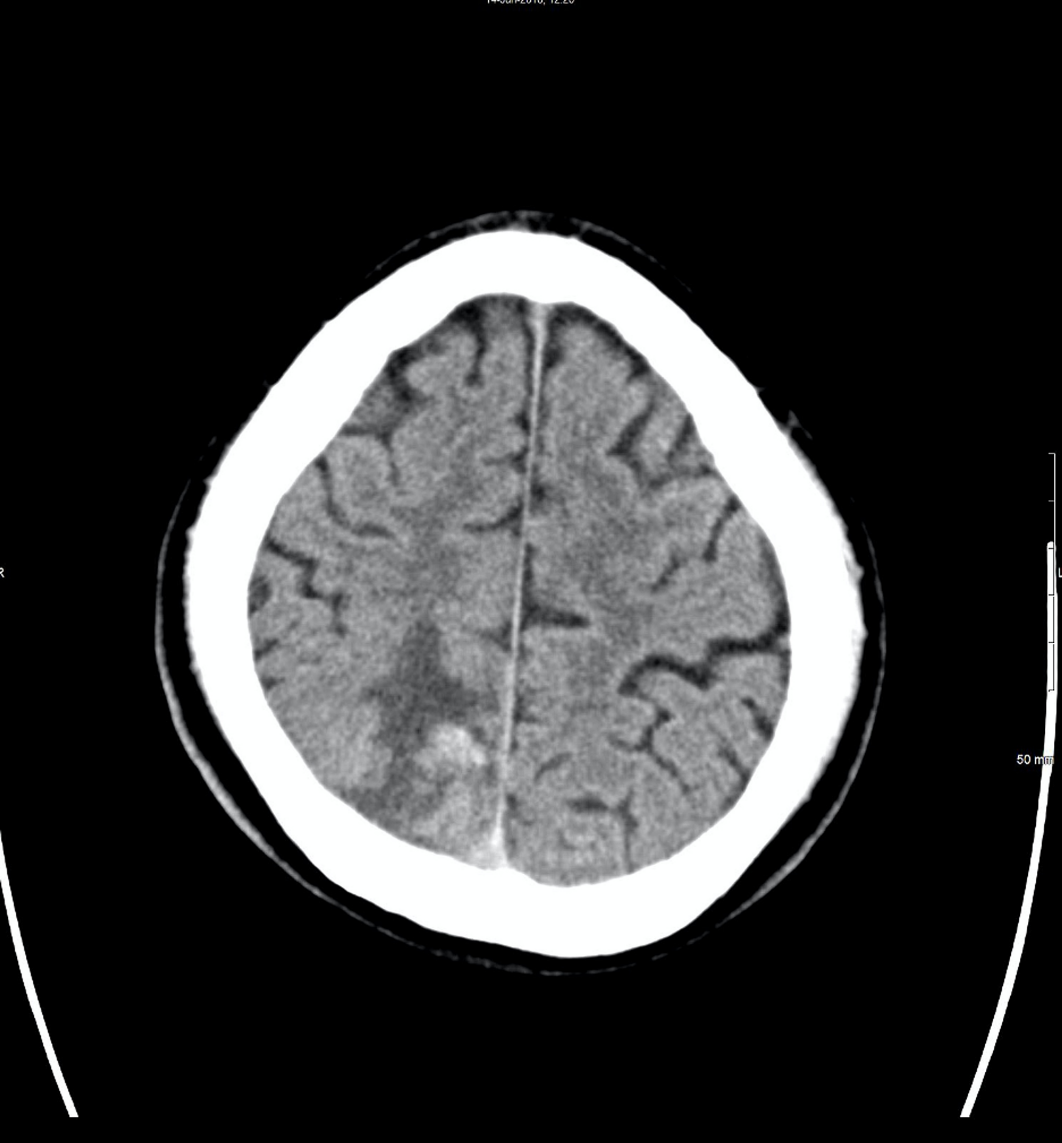
CT head, axial view. 1.0 cm hemorrhage at the gray-white junction of the posterior superior mesial right parietal lobe with surrounding edema.

**Figure 2:**
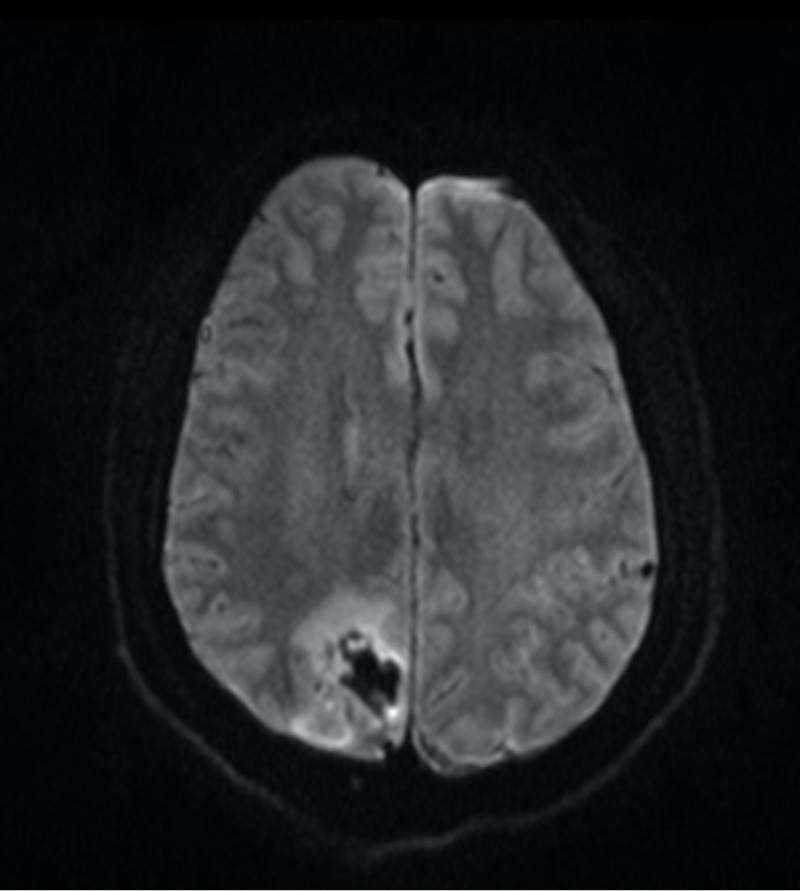
MRI brain, axial view. DWI with small areas of cortical restricted diffusion in the adjacent right paramedian posterior lobe.

**Figure 3:**
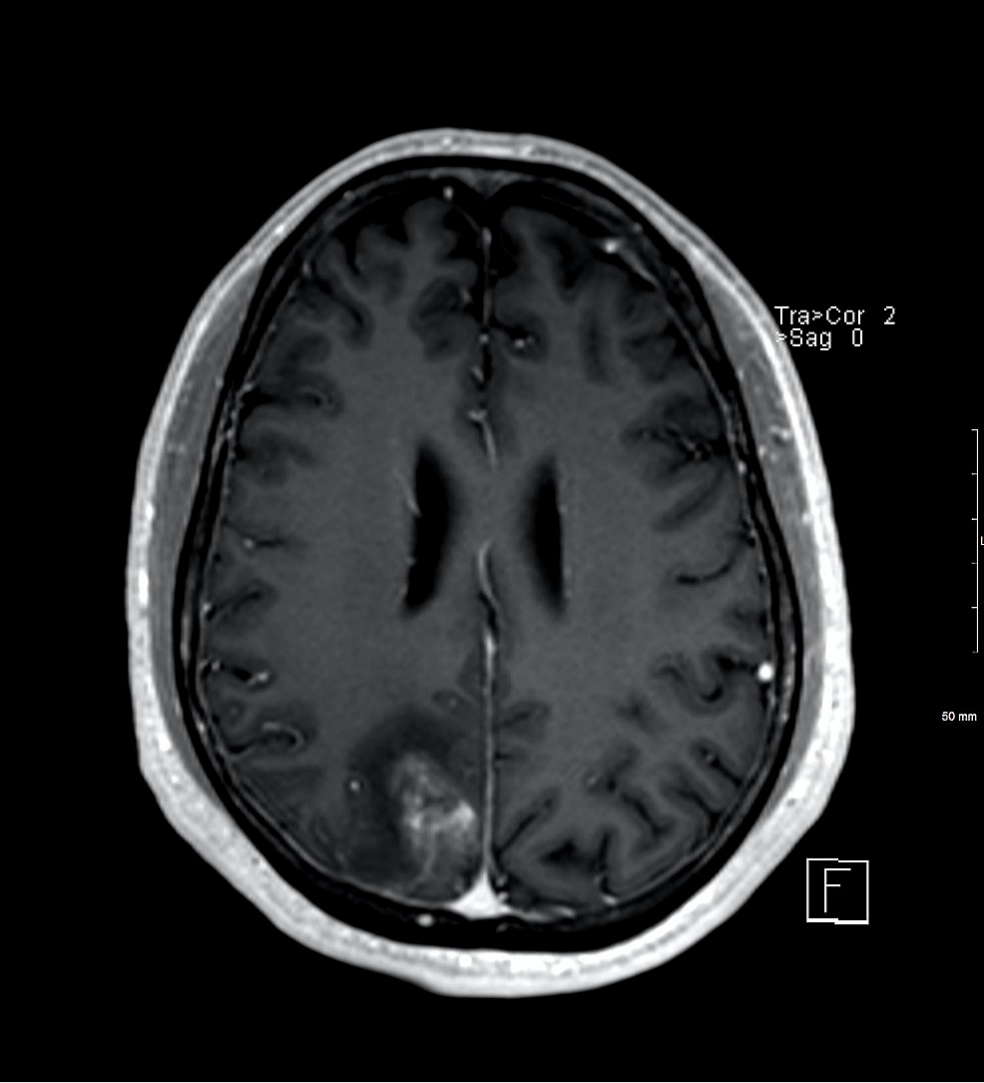
MRI brain with contrast, axial view. Hemorrhage surrounded by a small area of patchy enhancement in the right paramedian posterior lobe.

**Figure 4:**
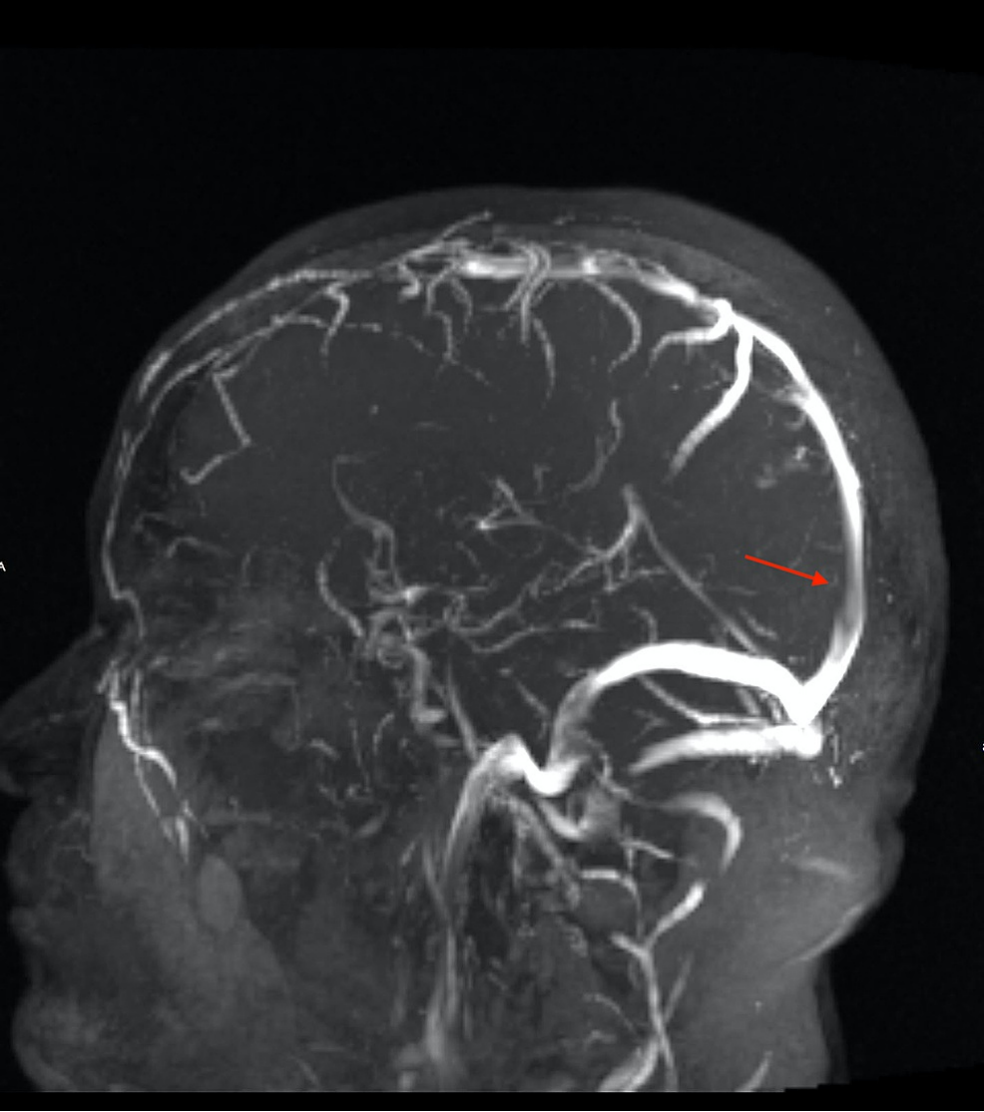
MRV brain, sagittal view. Irregularity and narrowing within the posterior aspect of the superior sagittal sinus.

Neurology was consulted for suspicion of CVA, and a stroke protocol was initiated. Computed tomography (CT) of the head was significant for a 1.0 centimeter (cm) hyperdense nodule at the posterior superior mesial right parietal lobe with surrounding edema, suggestive of a subacute cerebral infarct with hemorrhagic transformation (Figure 1). An MRI brain was completed next, confirming hemorrhage and vasogenic edema in the setting of an adjacent thrombosed cortical vein and partial thrombosis of the adjacent superior sagittal sinus (Figure 2, 3). Further, magnetic resonance venogram (MRV) confirmed a nonocclusive superior sagittal sinus thrombosis (Figure 4). Initial management included interventional neurology consult for cerebral angiogram, the commencement of a high-intensity statin, an initial hold on aspirin therapy due to hemorrhagic findings, and strict blood pressure control of 130/80 mmHg or less.

The following morning, cerebral angiogram demonstrated no large vessel occlusion, suggesting that MRI findings might be secondary to smaller vessel occlusions. After determining that the risk of further infarction was greater than the risk of worsening hemorrhage, the patient was started on a low dose heparin drip (16.6 units/kg/hr) with goal-activated partial thromboplastin time (aPTT) of 40-60 seconds. To monitor for worsening of hemorrhage while on anticoagulation, serial CTs of the head were ordered for the next 24 hours, all of which showed no hemorrhagic change.

To investigate the etiology of the CVST, a hypercoagulable workup was completed and was unremarkable. Negative results included tests for Factor V Leiden thrombophilia, Protein C and S deficiencies, antithrombin III deficiency, and rheumatologic etiologies. Repeat infectious markers showed a robust CD4 count of 827 cells/mm3, an undetectable viral load, no present opportunistic infections, and negative syphilis labs. The patient remained stable during his hospitalization with intermittent visual symptoms, but the improvement of headaches. He continued anticoagulation therapy with enoxaparin injections as a bridge to warfarin with a goal international normalized ratio (INR) of 2-3. He was discharged with a follow-up MRI brain scheduled six weeks later and outpatient colonoscopy for new-onset iron deficiency anemia.

**Figure 5:**
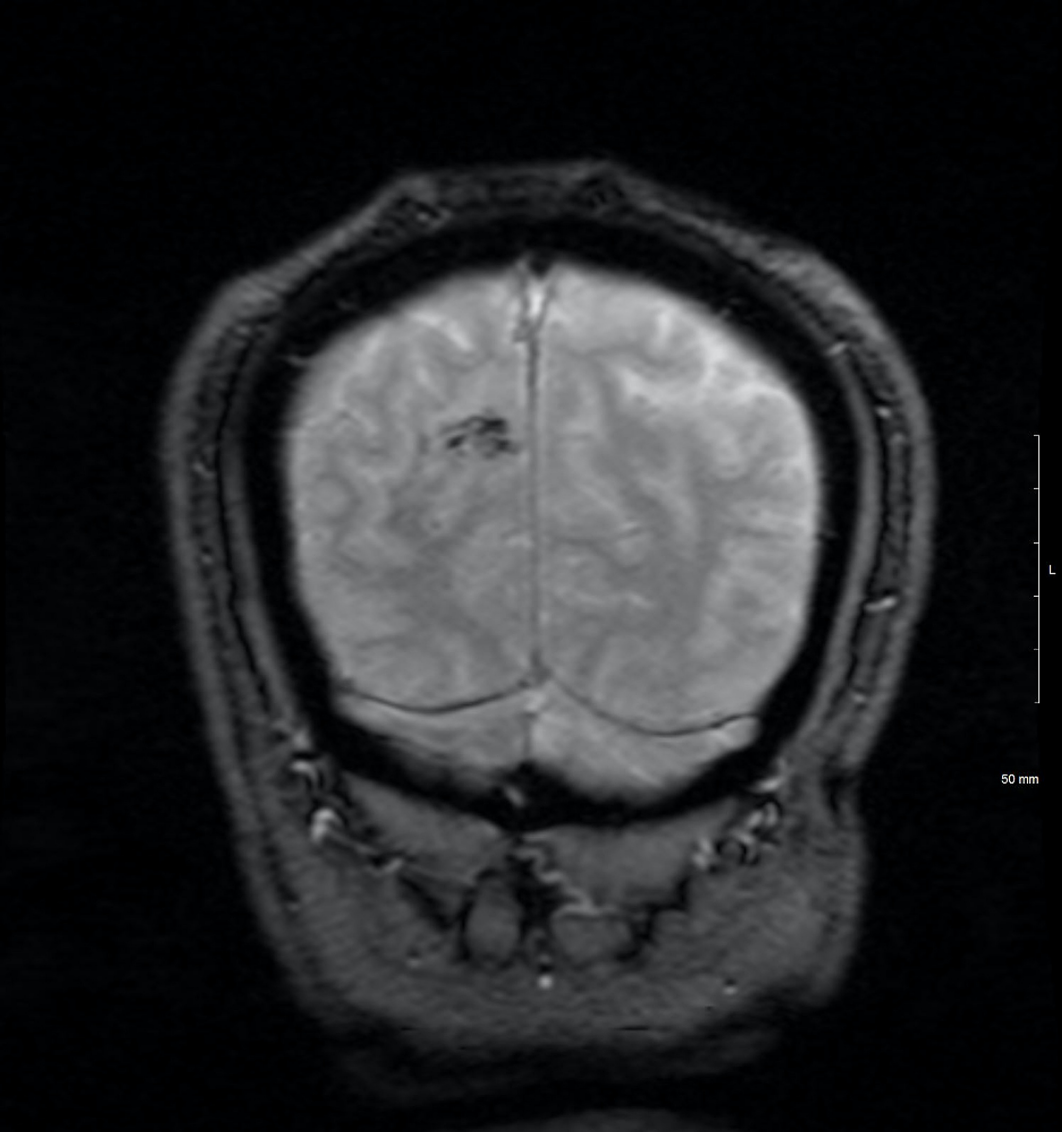
MRI brain, coronal view. T2 susceptibility weight images showing the evolution and decreased hemorrhage in the right posterior paramedian lobe.

At his two-month follow-up with neurology, the patient was stable and reported significant improvement in his symptoms. Iron supplementation had normalized his new-onset anemia, but no cause was identified. His MRI brain showed interval resolution of the T2 hyperintense signal in the adjacent cortical vein and superior sagittal sinus (Figure 5). When questioned most recently about any persistent symptoms, the patient admitted to ongoing migraines and new issues with remembering people’s names. However, he believed that his overall hospital treatment went well, without any major difficulties.

## Discussion

HIV promotes a prothrombotic state which predisposes patients to venous and arterial thromboembolism [1]. Endothelial injury caused by a primary HIV infection can also cause venous thromboses [2]. When the endothelial injury involves cerebrovasculature, cerebral infarction, intracranial hemorrhage, dural venous sinus thrombosis, and subdural hemorrhage may occur [2]. However, cerebrovascular thrombotic incidents such as CVSTs are less common as compared to systemic venous thromboses in HIV patients. In a single-center Netherlands retrospective study between 1989-2004, 519 HIV patients were hospitalized, with 19 patients (4%) suffering venous thromboses, where two of the 19 were CVSTs [3]. In summary, HIV predisposes patients to venous thromboses, but CVSTs occur rarely. It is therefore important to evaluate risk factors that contribute to further endothelial injury, vascular stasis, and hypercoagulability.

Both pro-coagulative and cardiovascular disease risk factors contribute to thrombotic insults in HIV patients. Factors that increase the likelihood of CVSTs include typical hypercoagulable states such as pregnancy and postpartum periods, oral contraceptive use, immobilization, obesity, and perioperative states [4,5,6]. Other risk factors including rheumatologic autoimmune disorders (i.e. systemic lupus erythematosus and antiphospholipid antibody syndrome), malignancy, and active infections may contribute to CVSTs [4,5,6]. Protein S, C, and antithrombin mutations are commonly reported in CVST, with several other hypercoagulable etiologies cited as well [4,5,6]. Additionally, a 2010 Danish study demonstrated that intravenous drug use severely increased the risk of VTEs in HIV patients [5]. While iron-deficient anemia has been shown to contribute to CVSTs in the pediatric population, there is less known about its association with CVST in adults [7].

Comprehensive laboratory investigation is recommended for all HIV patients with a CVST. Due to the breadth of underlying risk factors, workups should be tailored to address pertinent findings from the patient history and physical exam. However, a current CD4 count, complete blood count (CBC), comprehensive metabolic panel (CMP), and blood cultures should be at the core of the investigation [8]. Along with a viral load, hypercoagulability testing may be required. It is also necessary to inquire about previous and current opportunistic infections, tuberculosis (TB), and syphilis [1]. Our patient was overweight (190 pounds, BMI 28.89 kg/m^2^) but otherwise had no additional risk factors discovered in his medical history or laboratory workup which would further predispose him to his CVST. This suggests that despite his immunocompetence, the prothrombotic state of HIV was largely influential in his disease pathology.

Researchers have identified a significant association of CD4 counts and viral loads with the frequency of venous thromboses in HIV patients. In a single-center United States study of HIV patients between 1996-2007, those with low CD4 counts (median 153 cells/mm^3^) and high viral loads (3.64 log^10^ copies/mL) experienced increased rates of thrombosis occurrence. Each increase of 100 CD4 cells/mm^3^ was correlated with a 43% reduction in VTE risk [9]. A similar correlation between the occurrence of CVSTs, CD4 counts, and viral loads is yet to be established [5]. In a 2013 Botswana CVST case series in two HIV patients, one had a CD4 count above 200 cells/mm^3^, and the other had a CD4 count below 200 cells/mm^3^, with no documented viral loads [10]. Our patient presented with a CD4 count of 827 cells/mm^3^ and undetectable viral load. Together, these cases do not conform to the cell count and viral load trends seen in systemic venous thromboses, suggesting the need for further study of cell count and viral load correlation with CVST.

Multiple studies have investigated whether an association exists between highly active antiretroviral therapy (HAART), venous thrombosis, and stroke [9,11]. Protease inhibitors (PI) are hypothesized to cause platelet dysfunction and endothelium damage, thereby contributing to VTEs in HIV [9]. However, analysis has not produced conclusive results, indicating a weak correlation existing between HAART/PI usage and venous thromboses, if any at all [3,9]. Further data is necessary to establish a significant connection, and patients should continue HAART/PI as directed by their physician.

On presentation, patients will most frequently have a headache (90%), either of sudden onset or over the course of several days [6]. Patients may experience visual disturbances, such as blurred vision or complete vision loss [12]. Seizure is also seen (40%), with 50% of seizures being focal. Rarely, unilateral neurologic deficits such as hemiparesis or aphasia are reported [6]. Clinicians should begin with a CT head to evaluate for subarachnoid hemorrhage, depending on the acuity and severity of symptoms. Common findings on CT that may indicate CVST include cerebral edema, hemorrhagic bleeding, sinus band sign (20-30%), or empty sinus sign (δ sign) (16-46%) [13]. However, the CT head may be radiographically unremarkable. Heightened clinical suspicion, seizure, or unilateral neurological dysfunction would prompt further investigation with an MRI brain and possible MRV brain. MRI brain will show irregular hyperintensity at the area of thrombosis, with signal intensity dependent on the acuity of the thrombosis. MRV brain will characteristically demonstrate absent flow at the area of thrombosis. In conjunction, these modalities can accurately diagnose and locate CVSTs [6]. Most often, thromboses in the general population are detected within the transverse sinus (86.7%), sigmoid sinus (64.0%), and superior sagittal sinus (29.3%) [4]. Our patient did not deviate from the common presentation, as his MRI brain and MRV located a thrombosis within the superior sagittal sinus.

Although venous infarctions have the capacity to develop into hemorrhagic bleeds, anticoagulation is the accepted mainstay of CVST care. A 2012 Cochrane review demonstrated that heparin anticoagulation may reduce the risk of death or lifelong debilitation in patients with CVSTs by preventing further thromboses [10,14]. A 2012 guideline published by the Royal College of Physicians recommended patients begin a warfarin regimen with heparin bridging upon diagnosis and be followed with serial INRs [15]. Currently, no publications suggest whether direct oral anticoagulants, heparin, low molecular weight heparin, or warfarin have greater efficacy in preventing future thromboembolisms or death [8,16,17]. There also is no consensus as to the most beneficial duration of anticoagulation, but an international clinical trial is currently underway investigating outcomes in patients treated for six months and twelve months [18]. Local thrombolysis is another therapeutic option but requires extensive study before mainstream adoption. There is also a scarcity of information regarding anticoagulation regimen safety for HIV patients on HAART. Initially, warfarin was the de facto choice for HIV patients. However, discoveries of warfarin-drug interactions secondary to CYP450 augmentation have driven a renewed investigation into superior anticoagulant therapies. Direct oral anticoagulants are relatively safe and efficacious but suffer from interactions with HAART as well [5]. Through further anticoagulant research, HIV patients will hopefully benefit from advancements in therapy to come.

Stroke prevention strategies should be considered in HIV patients due to the increased risk for a CVA. Ischemic stroke is more common than hemorrhagic stroke in HIV patients, with hemorrhagic stroke frequency increasing proportionally with viral loads. Stroke risk factors in HIV are similar to that of CVST: the inherent prothrombotic state of HIV increased atherosclerotic changes with HAART, and coagulopathy due to opportunistic infections. HIV patients may also have traditional risk factors that increase their chance of stroke. HIV patients with cardiovascular disease have a higher risk of atrial fibrillation and atrial flutter, which can cause cardioembolic strokes [11]. Additionally, as advancements in HIV therapies have increased the life expectancy of patients, risk factors for stroke seen in the general population are increasing in prevalence in the HIV-positive community [1]. Therefore, early intervention and compliance with HAART for HIV, along with antiplatelets, antihypertensives, and statins for traditional risk factors are suggested for primary prevention. Through further assessment, the benefits of prevention methods may be more clearly understood, leading to therapeutic optimization.

In summary, HIV is not only an immunocompromising disease but a prothrombotic disorder. Due to its rare incidence, diagnosing CVST as a complication of HIV is difficult. However, identification of comorbid coagulative conditions may heighten clinical suspicion. Our case demonstrates the need for further analysis of how CD4 counts, viral loads, and HAART therapy impact the frequency of CVSTs in HIV patients. It also stresses the importance of diagnostic imaging and laboratory testing for appropriate management and resolution of this rare complication.

## Conclusions

In the setting of HIV, systemic VTEs are more frequent than their cerebral counterpart, CVSTs. HIV patients with low CD4 counts and high viral loads are more likely to suffer VTEs and CVSTs as compared to immunocompetent HIV patients. Therefore, our case of CVST in a patient with well-managed HIV is unique. Therapy and prognosis are similar to that of the general population when compared to those with HIV. Because this disease is able to affect almost every organ system in unique ways, it is imperative to keep a broad differential in the assessment of both HIV-positive patients with atypical disease presentations and patients of unknown HIV status. It is also necessary to be cognizant of HIV as an etiology in a patient presenting with VTE or CVST.
